# Supplementary material for: An olfactory self-test effectively screens for COVID-19
Source: Commun Med (Lond). 2022 Apr 5;2:34. doi: 10.1038/s43856-022-00095-7 (PMC9053292; doi:10.1038/s43856-022-00095-7)
Supplement: Supplementary file 8 — Supplementary Materials [file 43856_2022_95_MOESM8_ESM.pdf]

Supplementary Materials for:

An Olfactory Self-Test Effectively Screens for COVID-19

Kobi Snitz[1*], Danielle Honigstein[1*], Reut Weissgross[1*], Aharon Ravia[1*], Eva Mishor[1*], Ofer Perl[1*], Shiri Karagach[1], Abebe Medhanie[1], Nir Harel[2], Sagit Shushan[1,3], Yehudah Roth[3,4], Behzad Iravani[5], Artin Arshamian[5,6] Gernot Ernst[7], Masako Okamoto[8], Cindy Poo[9], Niccolò Bonacchi[9], Zachary Mainen[9], Erminio Monteleone[10], Caterina Dinnella[10], Sara Spinelli[10], Franklin Mariño-Sánchez[11], Camille Ferdenzi[12], Monique Smeets[13], Kazushige Touhara[8], Moustafa Bensafi[12], Thomas Hummel[14], Johan N. Lundström[5,15], Noam Sobel[1]

[1] Department of Neurobiology, Weizmann Institute of Science, Rehovot, Israel

[2] Bezalel Academy of Fine Arts and Design, Department of Fine Arts, Jerusalem, Israel

[3] Department of Otolaryngology- Head & Neck Surgery, Edith Wolfson Medical Center, Holon, Israel

[4] Sackler Faculty of Medicine, Tel Aviv University, Tel Aviv, Israel

[5] Department of Clinical Neuroscience, Karolinska Institutet, Stockholm, Sweden

[6] Department of Psychology, Stockholm University, Stockholm, Sweden

[7] Psychological institute, Oslo university, Oslo, Norway

[8] Department of Applied Biological Chemistry, Graduate School of Agricultural and Life Sciences, The University of Tokyo, Tokyo, Japan

[9] Champalimaud Research, Champalimaud Centre for the Unknown, Lisbon, Portugal

[10] Department of Agriculture, Food, Environment and Forestry, University of Florence, Florence, Italy

[11] Rhinology and Skull Base Surgery Unit. Otorhinolaryngology Department. Ramón y Cajal University Hospital. Madrid, Spain

[12] Lyon Neuroscience Research Center, CNRS - INSERM - University Claude Bernard of Lyon, Lyon, France

[13] Faculty of Social and Behavioral Sciences, Utrecht University, Utrecht, the Netherlands

[14] Smell and Taste Clinic, Department of Otorhinolaryngology, TU Dresden, Dresden, Germany

[15] Monell Chemical Senses Center, Philadelphia, Pennsylvania, USA

*These authors contributed equally

**Category 1**	**Category 2**	**Category 3**	**Category 4**	**Category 5**
Vanilla extract	Peanut butter	Mustard (Dijon)	Garlic (freshly chopped)	Your toothpaste
Nutella	Coconut oil	Vinegar (white)	Camembert cheese	Your hand soap
Honey	Olive oil	Horseradish (jar)	Canned tune	Your laundry detergent
Strawberry jam	Basil	Wasabi	Blue cheese	Your shampoo
Apricot jam	Oregano	Onion (freshly chopped)	Canned sardines	Your hand cream
Apple juice (not fresh)	Parsley	Vinegar (apple)	Mushrooms	Your body lotion
Orange juice (not fresh)	Cilantro	Black pepper (ground)	Boiled egg	Your perfume
Lemonade (not fresh)	Dill	Menthol gum	Pickled herring	Your hand sanitizer
Peach nectar (not fresh)	Cardamom	Mint (fresh)	Cumin	Your sunscreen
Pear nectar (not fresh)	Thyme	Mint (gum)	Soy sauce	Your baby oil
Grapefruit juice (not fresh)	Nutmeg	Mint (tea)	Sauerkraut	
Pineapple juice (not fresh)	Caraway	Sesame oil	Coffee (ground)	
Banana nectar (not fresh)	Bay leaves	Vodka	Coffee (instant)	
Cinnamon	Ketchup	Clove	Tea (black)	
Maple syrup		Vinegar (balsamic)	Tea (earl gray)	
		Vinegar (red)		
		Mustard (ordinary)		

Supplementary Table 1: The list of odorants participants could select from

Participants were instructed to select one odorant from each of the 5 categories, summing at 5 odorants for rating of intensity and pleasantness.
