# Supplementary material for: An olfactory self-test effectively screens for COVID-19
Source: Commun Med (Lond). 2022 Apr 5;2:34. doi: 10.1038/s43856-022-00095-7 (PMC9053292; doi:10.1038/s43856-022-00095-7)
Supplement: Supplementary file 9 — Description of Additional Supplementary Files [file 43856_2022_95_MOESM9_ESM.pdf]

Description of Additional Supplementary Files

File Name: Supplementary Data 1

Description: This file contains all the initial manuscript raw data

File Name: Supplementary Data 2

Description: This file contains all the manuscript raw data for the continuation (1464 participants)

File Name: Supplementary Data 3

Description: Source Data Figure 1

File Name: Supplementary Data 4

Description: Source Data Figure 2

File Name: Supplementary Data 5

Description: Source Data Figure 3

File Name: Supplementary Data 6

Description: Source Data Figure 4

File Name: Supplementary Data 7

Description: Source Data Figure 5
