# Supplementary material for: An olfactory self-test effectively screens for COVID-19
Source: Commun Med (Lond). 2022 Apr 5;2:34. doi: 10.1038/s43856-022-00095-7 (PMC9053292; doi:10.1038/s43856-022-00095-7)
Supplement: Supplementary file 11 — Reporting Summary [file 43856_2022_95_MOESM11_ESM.pdf]

nature research

Corresponding author(s): Noam Sobel

Last updated by author(s): Jan 23, 2022

Reporting Summary

Nature Research wishes to improve the reproducibility of the work that we publish. This form provides structure for consistency and transparency in reporting. For further information on Nature Research policies, see our Editorial Policies and the Editorial Policy Checklist.

Statistics

For all statistical analyses, confirm that the following items are present in the figure legend, table legend, main text, or Methods section.

n/a	Confirmed	
☐	☒	The exact sample size (*n*) for each experimental group/condition, given as a discrete number and unit of measurement
☐	☒	A statement on whether measurements were taken from distinct samples or whether the same sample was measured repeatedly
☐	☒	The statistical test(s) used AND whether they are one- or two-sided<br>*Only common tests should be described solely by name; describe more complex techniques in the Methods section.*
☐	☒	A description of all covariates tested
☐	☒	A description of any assumptions or corrections, such as tests of normality and adjustment for multiple comparisons
☐	☒	A full description of the statistical parameters including central tendency (e.g. means) or other basic estimates (e.g. regression coefficient) AND variation (e.g. standard deviation) or associated estimates of uncertainty (e.g. confidence intervals)
☐	☒	For null hypothesis testing, the test statistic (e.g. *F*, *t*, *r*) with confidence intervals, effect sizes, degrees of freedom and *P* value noted<br>*Give P values as exact values whenever suitable.*
☒	☐	For Bayesian analysis, information on the choice of priors and Markov chain Monte Carlo settings
☒	☐	For hierarchical and complex designs, identification of the appropriate level for tests and full reporting of outcomes
☐	☒	Estimates of effect sizes (e.g. Cohen's *d*, Pearson's *r*), indicating how they were calculated

Our web collection on statistics for biologists contains articles on many of the points above.

Software and code

Policy information about availability of computer code

Data collection	Data was collected online, using a website coded in open-source Drupal
Data analysis	All data analysis was with MATLAB (Release 2018b, 2019b, The MathWorks, Inc., Natick, Massachusetts, United States), and one extended data figure with Python (version 3.7.6). All analysis and figures code posted at https://gitlab.com/snitz/smelltracker_article, and also available for download at DOI: 10.5281/zenodo.5901547

For manuscripts utilizing custom algorithms or software that are central to the research but not yet described in published literature, software must be made available to editors and reviewers. We strongly encourage code deposition in a community repository (e.g. GitHub). See the Nature Research guidelines for submitting code & software for further information.

Data

Policy information about availability of data

All manuscripts must include a data availability statement. This statement should provide the following information, where applicable:

- Accession codes, unique identifiers, or web links for publicly available datasets
- A list of figures that have associated raw data
- A description of any restrictions on data availability

Data availability: All raw data are available for download in Supplementary Data Files 1 and 2. These files allow complete recreation of all figures accept the geographical mapping in Figure 1, as location data was stripped to protect privacy. All figure code also available at DOI: 10.5281/zenodo.5901547

Field-specific reporting

Please select the one below that is the best fit for your research. If you are not sure, read the appropriate sections before making your selection.

☐ Life sciences ☒ Behavioural & social sciences ☐ Ecological, evolutionary & environmental sciences

For a reference copy of the document with all sections, see nature.com/documents/nr-reporting-summary-flat.pdf

Behavioural & social sciences study design

All studies must disclose on these points even when the disclosure is negative.

Study description	This was an open on-line study. The study includes quantitative data that was obtained from participants ratings.
Research sample	Participants were self-selected, who ever logged on to the website participated. Overall we had 12020 participants (users) aged 18 and above.
Sampling strategy	There was no sampling strategy, but in analysis we randomly split the data 500 times
Data collection	This is an online study. Participants provided data alone in their homes, by using their computer mouse to mark a visual analogue scale (VAS) or select answers.
Timing	Between the dates of March 25th 2020 and September 23rd 2020.
Data exclusions	Only incomplete forms were excluded.
Non-participation	NA
Randomization	NA

Reporting for specific materials, systems and methods

We require information from authors about some types of materials, experimental systems and methods used in many studies. Here, indicate whether each material, system or method listed is relevant to your study. If you are not sure if a list item applies to your research, read the appropriate section before selecting a response.

Materials & experimental systems

n/a	Involved in the study	
☒	☐	Antibodies
☒	☐	Eukaryotic cell lines
☒	☐	Palaeontology and archaeology
☒	☐	Animals and other organisms
☐	☒	Human research participants
☒	☐	Clinical data
☒	☐	Dual use research of concern

Methods

n/a	Involved in the study	
☒	☐	ChIP-seq
☒	☐	Flow cytometry
☒	☐	MRI-based neuroimaging

Human research participants

Policy information about studies involving human research participants

Population characteristics	See above (Online participants self-selected, ages 18 and above)
Recruitment	No active recruitment. The web page was publicized in various media stories, and people logged on.
Ethics oversight	The study was approved by the Wolfson Hospital Helsinki Committee

Note that full information on the approval of the study protocol must also be provided in the manuscript.
